# Antibacterial properties and biological activity of 3D-printed titanium alloy implants with a near-infrared photoresponsive surface

**DOI:** 10.1186/s40729-024-00587-2

**Published:** 2025-01-08

**Authors:** Ming-Kang Wang, Fan Xiao, Xu Xu

**Affiliations:** 1 School of Stomatology, Zhejiang Chinese Medical University, Hangzhou, 310053 People’s Republic of China; 2 Department of Stomatology, The Quzhou Affiliated Hospital of Wenzhou Medical University, Quzhou People’s Hospital, Quzhou, 324000 People’s Republic of China; 3 College of Mechanical Engineering, Zhejiang University of Technology, 310023 Zhejiang, People’s Republic of China

**Keywords:** Ti6Al4V alloy, TiO_2_ surface modification, Near-infrared light, Photothermal antibacterial, Biocompatibility

## Abstract

**Purpose:**

SLM 3D printing technology is one of the most widely used implant-making technologies. However, the surfaces of the implants are relatively rough, and bacteria can easily adhere to them; increasing the risk of postoperative infection. Therefore, we prepared a near-infrared photoresponsive nano-TiO_2_ coating on the surface of an SLM 3D-printed titanium alloy sheet (Ti6Al4V) via a hydrothermal method to evaluate its antibacterial properties and biocompatibility.

**Methods:**

Using SLM technology, titanium alloy sheets were 3D printed, and a nano-TiO_2_ coating was prepared on its surface via a hydrothermal method to obtain Ti6Al4V@TiO_2_. The surface morphology, physicochemical properties, and photothermal response of the samples were observed. The Ti6Al4V groups and Ti6Al4V@TiO_2_ groups were cocultured with *S. aureus* and *E. coli* and exposed to 808 nm NIR light (0.8 W/cm^2^) and viable plate count experiments and live/dead bacterial staining were used to assess their in vitro antibacterial properties.

**Results:**

The hydrophilicity of the nano-TiO_2_ coating sample significantly improved and the sample exhibited an excellent photothermal response. The temperature reached 46.9± 0.32 °C after 15 min of irradiation with 808 nm NIR light (0.8 W/cm^2^). The Ti6Al4V group showed significant antibacterial properties after irradiation with 808 nm NIR light, and the Ti6Al4V@TiO_2_ group also had partial antibacterial ability without irradiation. After irradiation with 808 nm NIR light, the Ti6Al4V@TiO_2_ group showed the strongest antibacterial properties, reaching 90.11± 2.20% and 90.60± 1.08% against *S. aureus* and *E. coli*, respectively.

**Conclusions:**

A nano-TiO_2_ coating prepared via a hydrothermal method produced synergistic antibacterial effects after NIR light irradiation.

## Background

With the development of materials science and oral implant technology, dental implant restoration has gradually become one of the main methods to repair dentition defects. Titanium or titanium alloys are the main materials used for implants because of their excellent mechanical properties, corrosion resistance and biocompatibility [1, 2].

With the development of preparation technology, 3D printing is increasingly used in the processing and manufacturing of implants, and selective laser melting (SLM) is one of the most widely used technologies. SLM uses a high-energy laser beam to melt the metal powder according to the preset outline line, and the melted powder gradually accumulates into the finished three-dimensional metal parts [3]. The main advantages include high processing precision, a uniform finished product structure, and printing personalized implants matching alveolar sockets according to clinical needs [4].

However, the surfaces of both conventional implants and SLM 3D-printed implants are relatively rough; this rough surface is conducive to osseointegration but also promotes bacterial adhesion [5]. Hence, it is easy to cause postoperative complications, such as peri-implantitis. At present, the clinical treatment of peri-implantitis, such as mechanical therapy and antibiotic treatment, has problems such as failure to completely remove bacteria and can induce the production of drug-resistant bacteria. In contrast, photothermal antibacterial agents (PTAs) have gradually attracted the attention of researchers in recent years because of their lack of contact, low degree of side effects and efficient wide-spectrum antibacterial resistance [6, 7]. The term photothermal antibacterial (PTA) refers to a photothermal agent under the excitation of a specific light source, and the local temperature rapidly increases through the nonradiative relaxation of electrons to achieve an antibacterial effect [8]. Commonly used photothermal agents such as Ag, Cu, Fe, and polydopamine have been reported. The results prove that the above photothermal agents have a good photothermal antibacterial effect, and the antibacterial rate of *S. aureus*, *E. coli*, and other bacteria is close to 100% [9–12]. And low-energy light and mild thermal stimulation can promote osteoblast proliferation and differentiation [13, 14].

Previous studies have shown that nano-TiO_2_ has good photothermal properties, as well as good biocompatibility, mechanical properties, corrosion resistance [15], and some physical antibacterial ability [16, 17]. However, its wide band gap reaches 3.0–3.2 eV, requiring ultraviolet (UV) light activation [18] which can cause damage to the human body, greatly limiting its application. Therefore, we prepared a nano-TiO_2_ coating on the surface of SLM 3D-printed titanium alloy (Ti6Al4V) sheet via a hydrothermal method; this coating was activated using near-infrared (NIR) light at a wavelength of 808 nm to produce the photothermal properties so that the coating can further enhance its antibacterial ability through the photothermal effect on the basis of physical antibacterial. In addition, the nanostructures could promote the adhesion and proliferation of osteoblasts on the material surface [19, 20].

The purpose of this study was to examine the antibacterial effect of this nano-TiO_2_ coating under NIR light irradiation and investigate its biocompatibility. The hypothesis of this study was that the nano-TiO_2_ coating has photothermal properties and can exert an antibacterial effect and maintain biocompatibility under NIR light irradiation.

## Methods

### Preparation of the nano-TiO2 coating

SLM 3D-printed titanium alloy (Ti6Al4V) sheets with a diameter of 10 mm and a thickness of 1 mm were printed with a metal 3D printer (AM 400, Renishaw, UK) (processing parameters: power, 200 W; point spacing, 55 μm; exposure time, 50 s). The titanium alloy sheets were cleaned in ultrapure water for 15 min and pickled, the surface melted powder was removed, and the samples were sonicated with absolute ethanol and ultrapure water for 15 min before drying naturally. Then, the sample was placed in a 30% hydrogen peroxide solution, heated at 80 °C for 1 h, removed, cleaned with ultrapure water, and allowed to dry naturally. The titanium alloy sheet was placed into a Teflon-lined stainless-steel autoclave, and a precursor solution containing hexafluorotitanic acid (H_2_TiF_6_, 0.885 mM, Aladine), hydrochloric acid (HCl, 13 mM, Yonghua), isopropanol (C_3_H_8_O, 33 mM, Sinopharm), and hydrogen peroxide (H_2_O_2_, 13 mM, Yonghua) was added. After the reaction, the titanium alloy sheets were removed and cleaned with ultrapure water for 5 min; the experimental group was labelled Ti6Al4V@TiO_2_, and the control group did not undergo a hydrothermal reaction and was labelled Ti6Al4V. Both samples were placed in ultrapure water at 80 °C overnight and autoclaved at 120 °C for backup use.

### Materials characterization

The sample surface was observed via field-emission scanning electron microscopy (FE-SEM, SU 8000, Hitachi, Japan), and the surface elements were quantified by using energy dispersive X-ray spectrometry (EDS). The water contact angle was measured at room temperature with an optical contact angle metre (OCA 20, Dataphysic, Germany) to assess its hydrophilicity, and ultrapure water was used as the liquid (*n* = 5 per group). The crystal structure was identified via X-ray diffraction (XRD, X’Pert PRO, PANalytical) with a scan rate of 20°/min and a Cu-Kα source operated at 40 kV-40 mA.

### In vitro photothermal properties

#### Photothermal conversion capacity

The sample was immersed in 1 mL of phosphate-buffered saline (PBS) and irradiated with 808 nm NIR light (LSR808NL, Ningbo Yuanming, China) at a power of 0.8 W/cm^2^ for 15 min. The sample temperature was measured every 1 min with an infrared thermal imager, and the heating curve was plotted (*n* = 3 per group).

#### Photothermal stability

The sample was immersed in 1 mL of PBS and irradiated with 808 nm NIR light at a power of 0.8 W/cm^2^ for 15 min. Then, the light source was turned off, and the sample was naturally cooled for 15 min; this was considered one on‒off cycle. The sample temperature was measured every 1 min with an infrared thermal imager, and the temperature changes were repeated for three cycles.

### In vitro antibacterial properties

#### Viable plate count experiment

The experimental materials were divided into four groups and labelled Ti6Al4V, Ti6Al4V + NIR, Ti6Al4V@TiO_2_, and Ti6Al4V@TiO_2_ + NIR according to the presence or absence of illumination. The sterilized samples were placed in a 24-well plate, submerged with 1 mL S. aureus (ATCC 25923, People’s Hospital of Quzhou) / E. coli (ATCC 25922, People’s Hospital of Quzhou) suspension (1 × 10^6^ CFU/mL) and incubated a*t* 37 °C for 4 h. The samples were subsequently transferred to a new 24-well plate, 1 mL of PBS was added, and the illumination group was irradiated with NIR light at a wavelength of 808 nm at 0.8 W/cm^2^ for 15 min and rinsed with PBS to remove surface floating bacteria. The sample was placed in a centrifuge tube containing 5 mL of sterile PBS and shaken on a vortex oscillator for 5 min to shed bacteria from the sample surface into PBS. After the same multiple of dilutions for each group, 10 µL of bacterial suspension was evenly coated on agar plates and incubated at 37 °C for 24 h, the number of bacterial colonies was recorded, and the antibacterial rate of each group was calculated. The antibacterial rate = [(λ_0_ - λ_t_)/λ_0_] × 100%, where λ_0_ is the average number of viable bacteria in the Ti6Al4V group and λ_t_ is the average number of viable bacteria in the Ti6Al4V + NIR, Ti6Al4V@TiO_2_, and Ti6Al4V@TiO_2_ + NIR groups (*n* = 5 per group).

#### Live/dead bacterial staining

The sterilized samples were placed in a 24-well plate, submerged with 1 mL bacterial solution of 1 × 10^6^ CFU/mL, and incubated at 37 °C for 4 h. The samples were subsequently transferred to a new 24-well plate, 1 mL of PBS was added, and the illumination group was irradiated with NIR light at a wavelength of 808 nm at 0.8 W/cm^2^ for 15 min and rinsed with PBS to remove surface floating bacteria. In accordance with the instructions of the Live/Dead Bacteria Staining Kit (L13152, Thermo Fisher, USA), a mixture of SYTO 9 and propidium iodide (PI) was added to the sample surface, incubated for 15 min in a light-free environment. The samples were subsequently observed and photographed under a fluorescence inverted microscope. Images were fitted with ImageJ software (*n* = 5 per group).

### In vitro biocompatibility assessment

The Ti6Al4V group and the Ti6Al4V@TiO_2_ + NIR group were selected for the in vitro biocompatibility experiments based on the in vitro antimicrobial experimental results.

#### Culture of the osteoblasts

Mouse preosteoblasts (MC3T3-E1 Subclone 14, Propsy, China) were cultured in MC3T3-E1 Subclone 14 cell-specific medium (Propsy, China) at 37 °C and 5% CO_2_. The cells were digested with 0.25% trypsin (Yuanye, China) when they reached 90% confluence.

#### Cell adhesion

The sterilized samples were placed into a 24-well plate, the digested cells were inoculated onto the sample surface at a density of 2 × 10^4^ cells per well, and the illumination groups were irradiated with near-infrared light for 15 min. The samples were removed after 30, 60 and 120 min of culture, rinsed with PBS and placed in a new 24-well plate; next, 50 µL of CCK-8 solution (Solarbio, China) and 500 µL of cell culture medium were added to each well, and the samples were incubated in a 37 °C incubator for 2 h. 100 µL of the reaction mixture were added to a 96-well plate, and the OD at 450 nm was measured via a microplate reader (*n* = 5 per group).

#### Cell proliferation

The sterilized samples were placed into a 24-well plate, the digested cells were inoculated onto the sample surface at a density of 8000 cells per well, and the illumination groups were irradiated with near-infrared light for 15 min. Samples were removed after 1, 3, and 7 d of culture, rinsed with PBS and placed into a new 24-well plate, and 50 µL of CCK-8 solution and 500 µL of cell medium were added to each well; the well-plate was placed in a 37 °C incubator for 2 h. 100 µL of the reaction mixture were added to a 96-well plate, and the OD at 450 nm was measured via a microplate reader (*n* = 5 per group).

#### Cell morphology analysis

The sterilized samples were placed into a 24-well plate, the digested cells were inoculated onto the sample surface at a density of 8000 cells per well, and the illumination groups were irradiated with near-infrared light for 15 min. The samples were removed after 1, 3, and 7 d of culture; next, 2.5% glutaraldehyde (Yuanye, China) was added to the samples, and then they were placed in a 4 °C refrigerator overnight. The samples were rinsed with PBS and dehydrated with a gradient of 50%, 70%, 80%, 90%, and 100% ethanol. Afterward, tert-butanol was added to the samples, which were incubated at 4 °C for 10 min. The adhesion morphology of the cells on the sample surface was observed via SEM after drying and metal spraying (*n* = 5 per group).

#### Actin staining

The sterilized samples were placed into a 24-well plate, the digested cells were inoculated onto the sample surface at a density of 2 × 10^4^ cells per well, and the illumination groups were irradiated with near-infrared light for 15 min. The samples were removed after 2 and 6 h of incubation, rinsed with PBS, and fixed in 4% paraformaldehyde (Yuanye, China) for 20 min at room temperature. Then, the samples were treated with 0.5% Triton X-100 (Yuanye, China) for 5 min and rinsed with PBS. FITC phalloidine (Solarbio, China) was added, the sample was incubated for 30 min in a light-free environment. The cell nuclei were counterstained with DAPI dye solution for 3 min. Cell morphology and actin were observed under a fluorescence inverted microscope (*n* = 5 per group).

### Statistical analysis

Experimental data analysis was performed using SPSS 25.0 software. The results are presented as the mean ± standard deviation (SD). The Shapiro-Wilk test was used to assess the normality of variables and the Levene’s test was performed to assess the equality of variances. The results showed normal distribution and of the measurement values, and the datasets meet the assumptions of homoscedasticity. The differences between the groups were evaluated via t tests and one-way ANOVA followed by a least significant difference test. Statistical significance was accepted at ^*^p < 0.05.

## Results

### Sample characterization

**Fig. 1:**
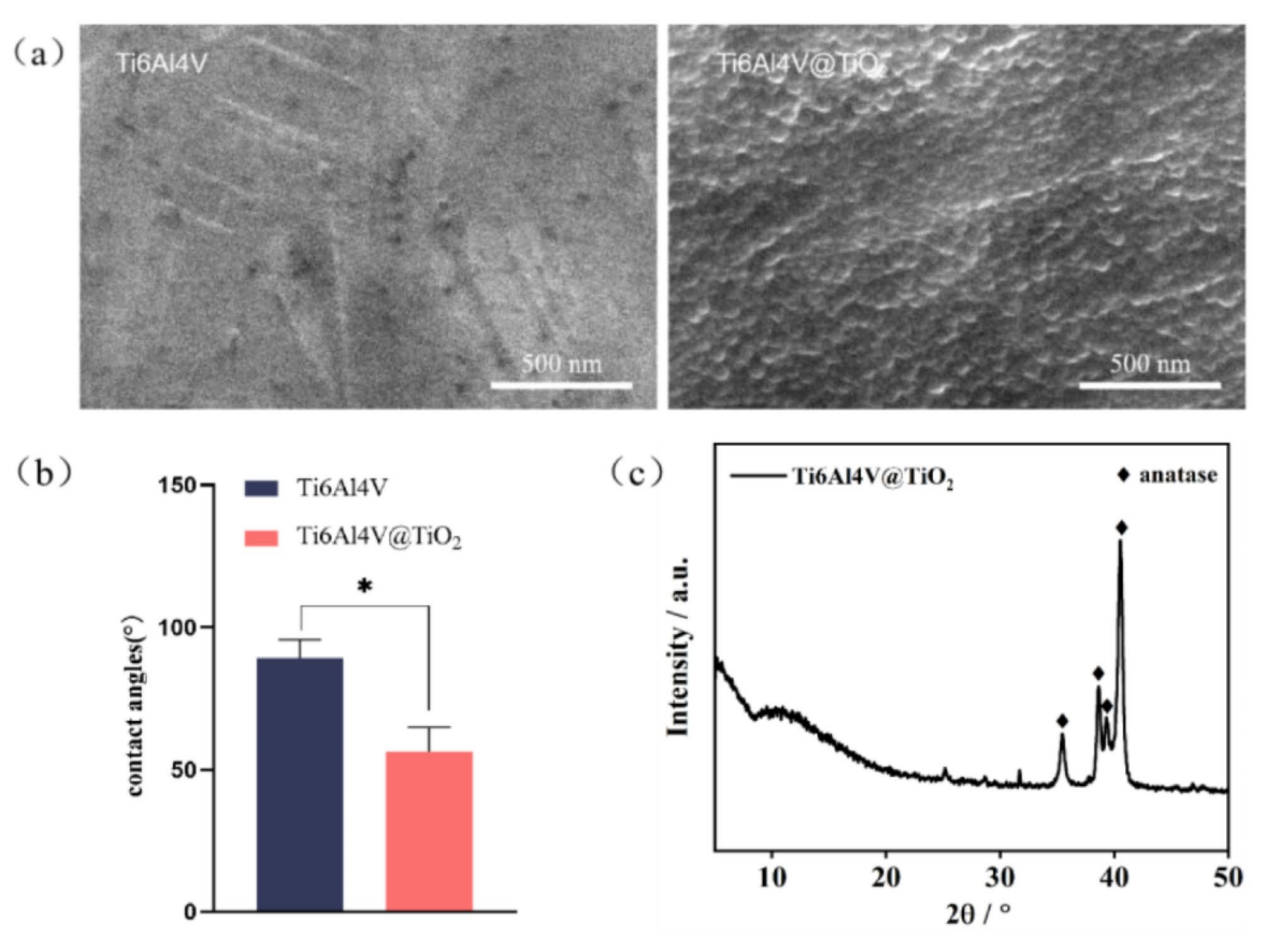
A highly hydrophilic nano-TiO_2_ coating was prepared on the Ti6Al4V surface with a hydrothermal method. (**a**) SEM images of the Ti6Al4V group and the Ti6Al4V@TiO_2_ groups; (**b**) Comparison of water contact angles between the Ti6Al4V and Ti6Al4V@TiO_2_ groups; (**c**) XRD patterns of the Ti6Al4V@TiO2 group

The surface morphology of the sample observed via SEM is shown in Fig. 1(a). The surface of the Ti6Al4V group was relatively flat, whereas the Ti6Al4V@TiO_2_ group formed a dense number of spherical nano-TiO_2_ particles. Based on the EDS data (Table 1), both groups contained Ti, Al and V, these elements were present in the Ti6Al4V matrix, and the proportion of O in the Ti6Al4V@TiO_2_ group was significantly greater than that in the Ti6Al4V group. As shown in Fig. 1(b), the water contact angle in the Ti6Al4V@TiO_2_ group was 56.3± 8.6°, which was less than the 89.2± 6.5° in the Ti6Al4V group, the difference was statistically significant (*P* < 0.05). The size of the contact angle is inversely proportional to the hydrophilicity of the material, thus the hydrophilicity of Ti6Al4V@TiO_2_ is significantly higher than Ti6Al4V. The XRD results are shown in Fig. 1(c), and the coating is mainly composed of TiO_2_ in the anatase phase.

**Table 1:** Chemical composition of samples (wt%)

Group	Ti	O	Al	V
Ti6Al4V Ti6Al4V@TiO_2_	70.56 54.67	19.88 38.63	7.16 4.44	2.40 2.26

### In vitro photothermal properties

**Fig. 2:**
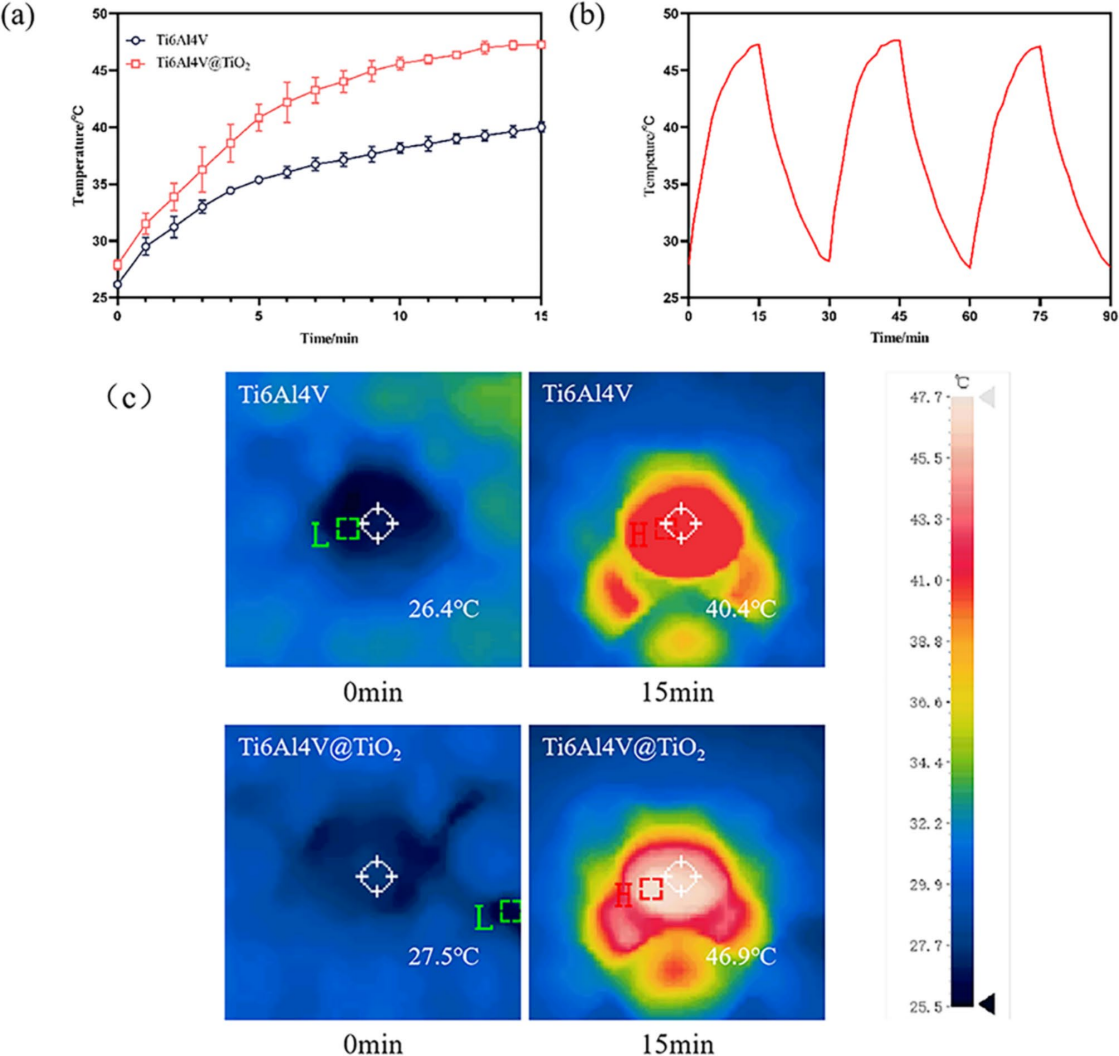
Nano-TiO_2_ coating has excellent photothermal conversion capability. (**a**) Temperature change of the sample in 1 mL of PBS under 808 nm NIR light (0.8 W/cm^2^) irradiation; (**b**) temperature change of the sample in 1 mL of PBS during on/off illumination (0.8 W/cm^2^); and (**c**) temperature measured with an infrared thermal imager

The heating curves from the two groups of samples under 0.8 W/cm^2^ power NIR light irradiation are shown in Fig. 2(a). As shown in Fig. 2(c), the final temperature of the Ti6Al4V group was stable at approximately 40.4± 0.44 °C, and the final temperature of the Ti6Al4V@TiO_2_ group was stable at approximately 46.9± 0.32 °C. The results of the photothermal stability experiments are shown in Fig. (b), with NIR light on or off the sample temperature presents a stable switching effect and the same phenomenon can be observed in later cycles.

### In vitro antibacterial properties

**Fig. 3:**
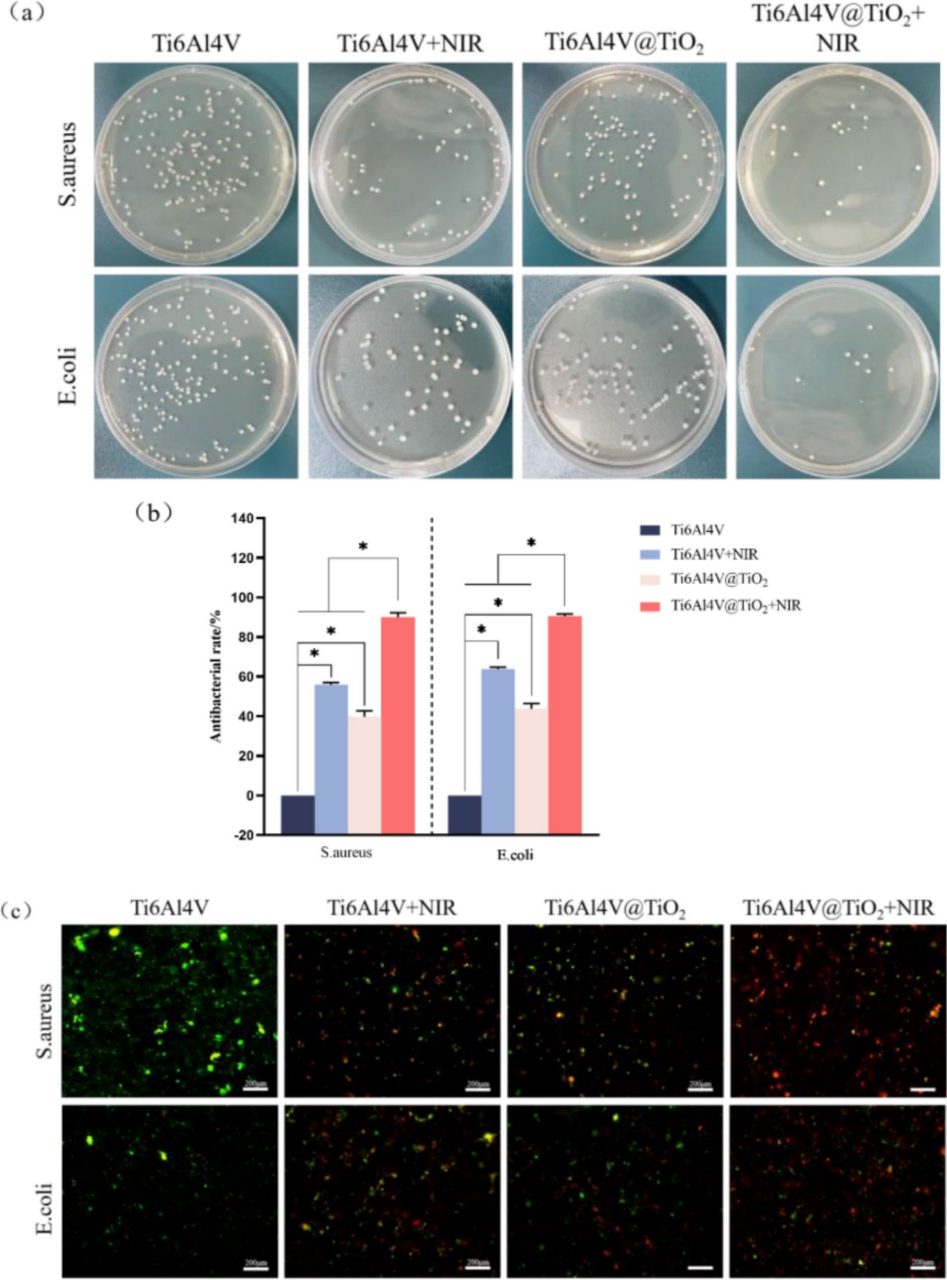
Nano-TiO_2_ coating had a significant antibacterial effect on S. aureus and E. coli after NIR irradiation. (**a**) Formation of bacterial colonies from different samples with or without 808 nm NIR light (0.8 W/cm^2^) irradiation; (**b**) antibacterial rate of different samples with or without 808 nm NIR light (0.8 W/cm^2^) irradiation; and (**c**) status of live/dead bacterial staining of different samples with or without 808 nm NIR light (0.8 W/cm^2^) irradiation

The results of the plate viable bacteria count experiment were as shown in Fig. 3 (a and b), the remaining three groups had significantly stronger antimicrobial rates compared to the Ti6Al4V group (*P* < 0.05). The antimicrobial rate in the Ti6Al4V + NIR group was significantly higher than that in the remaining three groups (*P* < 0.05), reaching 90.11± 2.20% and 90.60± 1.08% for S. aureus and E. coli, respectively. The results from the live/dead bacterial staining are shown in Fig. 3(c). SYTO 9 is a green fluorescent nucleic acid stain that can penetrate the cell membrane of live bacteria and dead bacteria. PI is a red fluorescent nucleic acid stain that can penetrate only the cell membrane of bacteria; both stains are simultaneously used, live bacteria have green fluorescence, and dead bacteria have red fluorescence. A large amount of green fluorescence was visible on the surface of the Ti6Al4V group, indicating that the bacteria adhered extensively on the surface. In the Ti6Al4V + NIR group and Ti6Al4V@TiO_2_ group, the surface green fluorescence significantly decreased, and the red fluorescence increased; these results indicate that both the Ti6Al4V + NIR group and the Ti6Al4V@TiO_2_ group had antibacterial effects. A large range of red fluorescence on the surface of the Ti6Al4V@TiO_2_ + NIR group was observed, and the surface bacteria were almost eliminated; thus, this group had the strongest antibacterial properties.

### In vitro biocompatibility

**Fig. 4:**
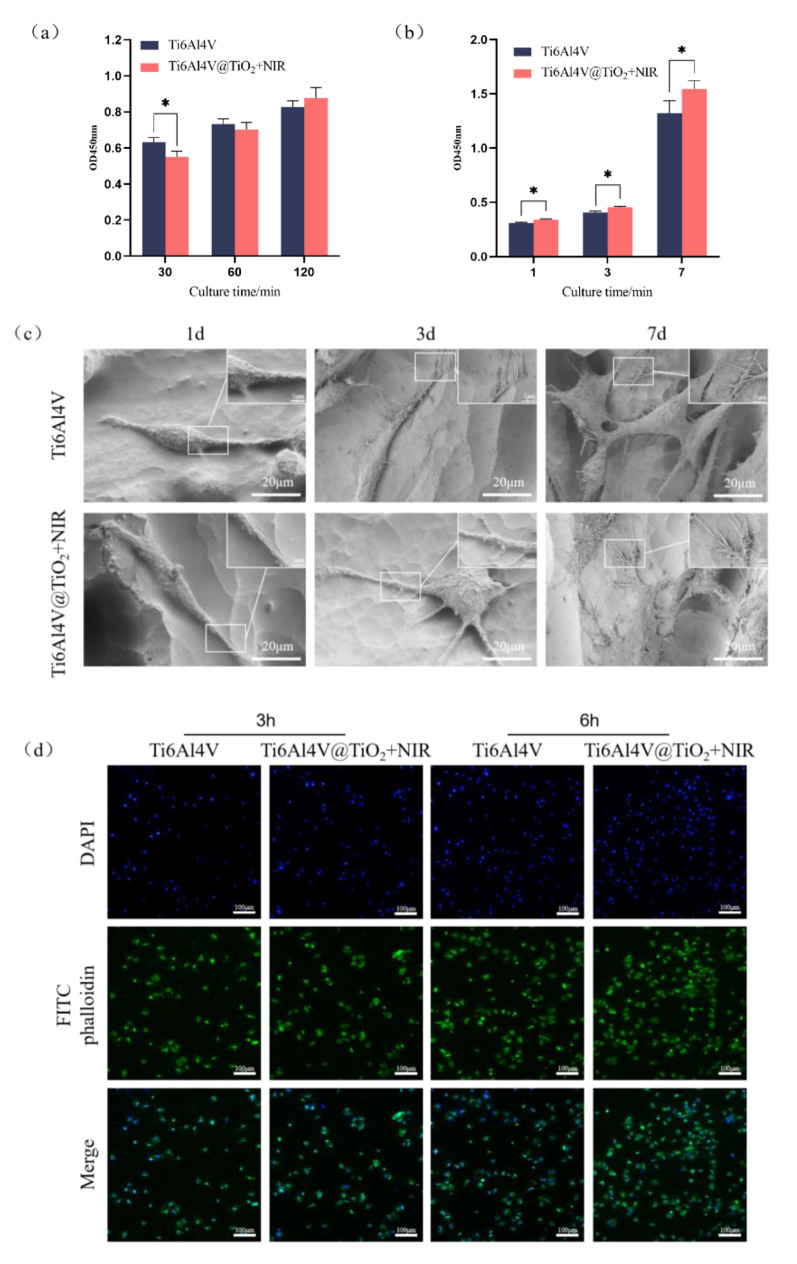
There were no significant adverse effects on the MC3T3-E1 osteoblasts on the nano-TiO_2_ coating surface after NIR irradiation. (**a**) Cell adhesion measured by CCK-8 after culture for 30, 60, or 120 min with or without 808 nm NIR light (0.8 W/cm^2^) irradiation; (**b**) cell proliferation measured by CCK-8 after culturing for 1, 3, and 5 d with or without 808 nm NIR light (0.8 W/cm^2^) irradiation; (**c**) SEM images of the MC3T3-E1 osteoblasts on different samples with or without 808 nm NIR light (0.8 W/cm^2^) irradiation; and (**d**) fluorescence staining images of the MC3T3-E1 osteoblast nuclei and actin with or without 808 nm NIR light (0.8 W/cm^2^) irradiation

Based on the results of the in vitro antibacterial experiments, we selected the Ti6Al4V@TiO_2_ + NIR group with the strongest antibacterial performance to test its in vitro biocompatibility and used Ti6Al4V as the control group to evaluate whether Ti6Al4V@TiO_2_ would cause damage to cells after NIR light irradiation. The CCK-8 assay was used to assess the effect of Ti6Al4V@TiO_2_ on the early adhesion of MC3T3-E1 osteoblasts after 15 min of exposure to NIR light, and the Ti6Al4V group served the control group. As shown in Fig. 4(a), at 30 min in culture, the Ti6Al4V@TiO_2_ + NIR group had significantly fewer surface adhesive cells than the Ti6Al4V group (*P* < 0.05). However, after 60 min and 120 min of culture, no significant difference in the cell adhesion capacity was observed between the Ti6Al4V@TiO_2_ + NIR and Ti6Al4V groups (*P* > 0.05).

Figure 4(b) shows the proliferation of MC3T3-E1 osteoblast cultures after 1, 3, and 7 d via the CCK-8 method. At 1, 3, and 7 d, the cell proliferation was significantly greater in the Ti6Al4V@TiO_2_ + NIR group than in the Ti6Al4V group (*P* < 0.05).

The adhesive morphology of MC3T3-E1 osteoblasts was also examined via SEM during culture on the sample surfaces for 1, 3, and 7 d. The results are shown in Fig. 4(c). At 1 d, no evident difference was observed in the surface osteoblast morphology between the Ti6Al4V and Ti6Al4V@TiO_2_ + NIR groups; this presented a spindle pattern with fewer and shorter pseudopodia. At 3 d, the surface cells of the Ti6Al4V group were long, spindle shaped and elongated, whereas those of the Ti6Al4V@TiO_2_ + NIR group were significantly spread, with elongated pseudo-feet and more antennae. At 7 d, more cells could be observed on the surface of the Ti6Al4V group, with spread polygons and many pseudopodia, and the cells were connected to each other. Compared to the Ti6Al4V group, the Ti6Al4V@TiO_2_ + NIR group had more cells on the surface, with better spread morphology and closer intercellular connections. The adhesive morphology of the cells on the surface of a material is one of the important factors for the success of material implantation.

Figure 4(d) shows the focal adhesion extension morphology of the MC3T3-E1 osteoblasts on the surface of the sample after fluorescence staining. The results showed that after 3 h of culture, the cells of Ti6Al4V and Ti6Al4V@TiO_2_ + NIR groups showed no evident difference in morphology, and some cells had extended short pseudopodia, with no significant elongation of actin. After 6 h of culture, the number of cells in the Ti6Al4V group increased, the cell morphology improved, the cells were polygonal, and the actin began to elongate. The Ti6Al4V@TiO_2_ + NIR group had a greater number of cells, a better cell spread morphology and cells protruding lamellipodia, the actin was more elongated and the actin fibres were clearer.

## Discussion

The biological cause of peri-implantitis formation is that a variety of bacteria attach to the implant surface to form a bacterial biofilm, leading to peri-implant infection and developing inflammation. PTA has been shown to have highly effective antimicrobial properties and can reduce the inflammatory response [21]. Good osseointegration is fundamental to the success of dental implants [22], so that during the surface modification of the implants, cannot damage the biological activity of the material itself. In this study, we investigated the photothermal properties, photothermal antibacterial capability, as well as the biological activity of nano-TiO_2_ coatings on the surface of titanium alloys.

A series of experiments on material characterization indicated the formation of an oxide layer on the sample surface and the nano-TiO_2_ coating was successfully prepared on the sample surface using the hydrothermal method. The water contact angle is closely related to the hydrophilicity of the material surface, and the water contact angle of highly hydrophilic materials is smaller. The water contact angle in the Ti6Al4V@TiO_2_ group was less than the Ti6Al4V group, indicating that the formation of nano-TiO_2_ improved the hydrophilicity of the material, potentially related to the larger surface area of the nanostructure. Most current views suggest that the surface of highly hydrophilic materials can promote the adhesion of fibronectin thus promoting the adhesion of osteoblasts [23].

To assess the in vitro photothermal conversion ability of the nano-TiO_2_ coating, we irradiated the samples in PBS with 808 nm NIR light and measured their temperature every minute. Numerous studies have shown that if the temperature of the implant socket exceeds 47 °C, then the osseointegration of the implant will be damaged and the probability of implant failure will increase significantly, so the critical temperature of the implant is set to 47 °C [24]. Therefore, we adjusted the irradiation power of the NIR light to ensure that the stable temperature did not exceed 47 °C and determined that the optimal NIR optical power was 0.8 W/cm^2^. The temperature of the sample increased with illumination time, eventually reaching a plateau. The final temperature of the Ti6Al4V group was stable at approximately 40.4 °C, and the final temperature of the Ti6Al4V@TiO_2_ group was stable at approximately 46.9 °C. The nano-TiO_2_ coating had a good photothermal conversion effect because of its inherent optical properties and its nanostructure [25]. At the same time, the photothermal stability experiment shows that nano TiO_2_ coating has excellent photothermal stability, can withstand repeated illumination and play an antibacterial effect, which is particularly important for clinical use.

Due to the complex microbial environment in the mouth, preventing infection after implantation is particularly important. In this study, we selected representative gram-positive and gram-negative bacteria: *S. aureus* and *E. coli*. Using Ti6Al4V as a control, the antimicrobial ability of each group was assessed using viable plate count experiments. The three groups produced significant antimicrobial effects on both bacteria; among them, the antibacterial effects of the Ti6Al4V + NIR group against *S. aureus* and *E. coli* were approximately 55.83% and 63.84%, respectively. This potentially occurred because Ti6Al4V also has partial photothermal antibacterial ability under irradiation with NIR light, as described above, and the temperature could reach 40.4 °C after 15 min of irradiation. However, the resistance of bacteria to sudden increases in temperature was much weaker than that to direct heating. At this time, the bacterial cell membrane ruptured, causing changes in permeability and the degeneration of the bacterial proteins, which eventually led to the death of the bacteria [26]. The antibacterial rates of the Ti6Al4V@TiO_2_ group against *S. aureus* and *E. coli* were approximately 39.70% and 43.78%, respectively, which could be attributed to the inherent antimicrobial properties of the nanosurface [27]. Bhardwaj and Webster [28] reported that a nanoscale TiO_2_ coating could affect the adsorption of proteins by changing the surface free energy to achieve bacteriostatic effects. Jenkins et al. [29] showed that nanostructures could destroy the bacterial cell membrane by stretching and piercing the bacterial cell membrane, thus killing bacteria. The Ti6Al4V@TiO_2_ + NIR group had the strongest antibacterial ability, and the antibacterial rates reached 90.11± 2.20% and 90.60± 1.08% for *S. aureus* and *E. coli*, respectively. Thus, due to the excellent photothermal properties of the nano-TiO_2_ coating and its physical antibacterial properties, Ti6Al4V@TiO_2_ could eliminate most of the bacteria within 15 min under 0.8 W/cm^2^ NIR light irradiation. A comparison of *S. aureus* and *E. coli* revealed that the antibacterial effect of each group against *S. aureus* was weaker than that against E. aureus, probably because *S. aureus* is a gram-positive bacterium with thicker bacterial cell walls, whereas *E. coli* has a thin cell wall; thus, relative to *S. aureus*, *E. coli* is more sensitive to temperature changes and the surface of the nanostructures [30]. The results of the live/dead bacterial staining correspond to the results of the viable plate count experiment, providing further confirmation.

The CCK-method was used to assess whether Ti6Al4V@TiO_2_ caused damage to cells after NIR irradiation. The results suggest that after light exposure early osteoblast adhesion is partially affected, but there is quick recovery from these effects. This likely occurred because the photothermal effect of NIR light irradiation at 0.8 W/cm^2^ had some effect on the cell adhesion ability, but this effect could be eliminated in the short term. On the one hand, Ti6Al4V@TiO_2_ has smaller water contact angles, with better hydrophilic properties, and the improved hydrophilicity could promote cell adhesion to the surface of the material [31]. On the other hand, the nano-TiO_2_ coating changed the roughness of the material surface for more favourable cell adhesion. Studies have shown that when the surface roughness of an implant is close to that of bone tissue, the implant can strongly promote the adhesion of osteoblasts [32, 33]. At 1, 3 and 7 d, the proliferative capacity of Ti6Al4V@TiO_2_ surface cells was not affected by NIR illumination; instead, they were significantly better than the control cells. This was caused by the hydrophilic surface of Ti6Al4V@TiO_2_ and the rough surface at the nanoscale. High hydrophilicity and rough surfaces could promote the proliferation of osteoblasts [34].

The adhesive morphology of cells on the material surface is one of the important factors for the success of material implantation. The SEM results revealed that the number and spread of surface cells in the Ti6Al4V@TiO_2_ + NIR group were greater than those in the Ti6Al4V group; thus, the promotion of the adhesion capacity of MC3T3-E1 osteoblasts by the nano-TiO_2_ coating was sufficient to eliminate the photothermal effect produced by the NIR irradiation of the nano-TiO_2_ coating on early cell adhesion. Studies have shown that cells on nanostructured TiO_2_ surfaces have abundant filopodia anchored to the material surface, thereby promoting cell adhesion. The mechanism involves mainly the cell alignment of the cytoskeleton and the formation of focal adhesions [35–37].

These results of actin staining were consistent with those from the cell adhesion capacity assessed by the CCK-8 assay. The nano-TiO_2_ coating could promote cell extension and thus adhesion; these results further indicated that the side effects of NIR light irradiation on cell behaviour were negligible. Nanostructures have been shown to promote cytoskeletal extension by promoting the expression of integrins and focal adhesion spot formation, thus promoting cell adhesion [38].

This study did have some limitations. First, the pathogenic bacteria of peri-implantitis are mostly anaerobic bacteria such as P.gingivalis and F.nucleatum, but this study was limited to S.aureus and E.coli. The inhibitory effect of nano-TiO_2_ coating on various bacteria needs to be further investigated. In addition, we lack the relevant in vivo experiments to further validate the results of our study.

## Conclusion

In conclusion, we prepared nano-TiO_2_ coatings on Ti6Al4V surfaces via a hydrothermal method, and the resulting coatings had a good photothermal response. With 808 nm NIR light irradiation, we established a synergistic antimicrobial model. The antibacterial mechanisms mainly included the photothermal effect of nano-TiO_2_ coating by NIR light irradiation and the destruction of the bacterial cell membrane by nanostructures. After NIR light (0.8 W/cm^2^) irradiation of the Ti6Al4V@TiO_2_ sample, the adhesion ability of MC3T3-E1 osteoblasts in the early stage was partially affected, but this effect was quickly eliminated; due to the nanosurface of Ti6Al4V@TiO_2_, the adhesion and proliferation ability of cells were further improved. This study provides a highly effective antibacterial strategy to reduce a series of postimplant complications due to infection and to ensure improved biological safety, convenient application and low cost.

## Data Availability

No datasets were generated or analysed during the current study.

## Abbreviations

S. aureus: Staphylococcus aureus
E. coli: Escherichia coli
CFU: Colony forming units
CCK-8: Cell Counting Kit-8
OD: Optical density
